# Transmission matrix of a multimode fiber: In-line vs off-axis holography

**DOI:** 10.1371/journal.pone.0340823

**Published:** 2026-02-06

**Authors:** Aleksandra Ivanina, Benjamin Lochocki, Andreas Koglbauer, Sergei Sokolov, Sebastianus A. Goorden, Lyubov V. Amitonova

**Affiliations:** 1 Advanced Research Center for Nanolithography (ARCNL), Amsterdam, The Netherlands; 2 Vrije Universiteit Amsterdam (VU), Department of Physics and Astronomy, Amsterdam, The Netherlands; 3 SCHOTT AG, Mainz, Germany; 4 ASML Research, Veldhoven, The Netherlands; Portland State University, UNITED STATES OF AMERICA

## Abstract

Multimode fibers (MMFs) hold great promise for a variety of imaging applications. However, controlling the complex propagation of light through thousands of modes in MMFs remains a challenging task. To enable precise wavefront control, the MMF transmission matrix (TM) must be accurately measured. In this paper, we compare in-line and off-axis holography as phase retrieval techniques for TM measurement within a single optical setup. The quality of wavefront shaping is analyzed for different target shapes, including single-mode and complex free-form projections. Our findings reveal a trade-off in wavefront shaping: while in-line holography achieves superior focus quality for single-mode targets, off-axis holography excels in projecting complex-shaped targets. These experimental findings provide a basis for choosing and optimizing holographic techniques in MMF-based imaging setups.

## Introduction

In recent years, there has been increasing interest in multimode fibers (MMFs) as a promising tool for optical communication, optical trapping, optical metrology, and endoscopic imaging [1,2]. The main challenge in MMF imaging is the complex propagation of light through thousands of interfering modes, which makes reliable information transmission difficult [3]. To address this, researchers have developed techniques such as conventional wavefront shaping [4] and digital phase conjugation [5–8], enabling the manipulation of the light’s spatial, temporal, and polarization degrees of freedom to optimize the fiber’s output [9–11]. Furthermore, accurately characterizing the scattering properties of complex media is crucial for imaging through turbid samples [12].

A complete solution that enables full control over light propagation through complex media is to measure a full transmission matrix (TM) of the MMF [13–16]. TM is a complex-valued quantity that contains both amplitude and phase information. Therefore, the choice of phase retrieval method directly affects the accuracy of the TM measurement and the effectiveness of subsequent light manipulation. Interferometric approaches such as in-line [17–21] and off-axis holography [22–27] are commonly used to retrieve phase information. These methods generally have their pros and cons: in-line holography offers a simple and stable setup but can suffer from blind spots [28] due to the speckled reference, while off-axis holography avoids these artifacts at the cost of increased system complexity and sensitivity to phase drift. Active stabilization techniques with feedback control systems or computational methods may be necessary to compensate for phase drift [22,23,29].

Several studies have compared these approaches. Gross et al. [30] imaged a USAF resolution target using both methods and found that off-axis enables fast, single-shot acquisition but suffers from aliasing, while in-line removes unwanted terms and produces cleaner target projections. Turtaev et al. [31] compared both methods for TM-based light control using liquid crystal spatial light modulators (LC-SLMs) and digital micromirror devices (DMDs), to generate focal spots. They reported that DMDs, used with a common-path reference, achieved higher measurement speed and better focusing fidelity than LC-SLMs, which were tested with an off-axis reference. The common-path geometry also improved stability. Cižmár et al. [16] demonstrated both focal spot and complex pattern projection at the MMF output using a measured TM with the Gerchberg-Saxton algorithm, relying on iterative computational phase retrieval.

In this work, we experimentally compare two interferometric phase retrieval approaches: in-line and off-axis holography for TM measurements of an MMF, within the same DMD-based setup. In contrast to previous studies, we investigate their performance for projecting both single focal spots and complex-shaped illumination patterns. We found that off-axis holography delivers higher fidelity for complex-shape projections. In contrast, in-line holography is more robust and stable for single-spot focusing due to its common-path geometry, which reduces sensitivity to phase drift. These findings provide practical guidance for selecting an interferometric phase retrieval method in MMF-based imaging systems depending on the application requirements.

## Methods

### Transmission matrix

The TM is a mathematical representation of how light propagates through complex media [13]. Although in our experiments light propagates through guided fiber modes, we measure and use the TM in the spatial basis, where the input modes *n* correspond to discrete spatial segments on the DMD, and the output modes *m* correspond to pixels on the output camera. This basis aligns with the experimental degrees of control and observation, allowing us to express the light propagation as:

$$E_{m}^{out}=\sum_{n}k_{mn}E_{n}^{in},$$ (1)

where $E_{n}^{in}$ denotes the complex field modulated by the *n*-th DMD segment, $E_{m}^{out}$ is the complex field measured at the *m*-th camera pixel, and *k*_*mn*_ are the complex-valued elements of the TM.

### Experimental setup

**Fig 1:**
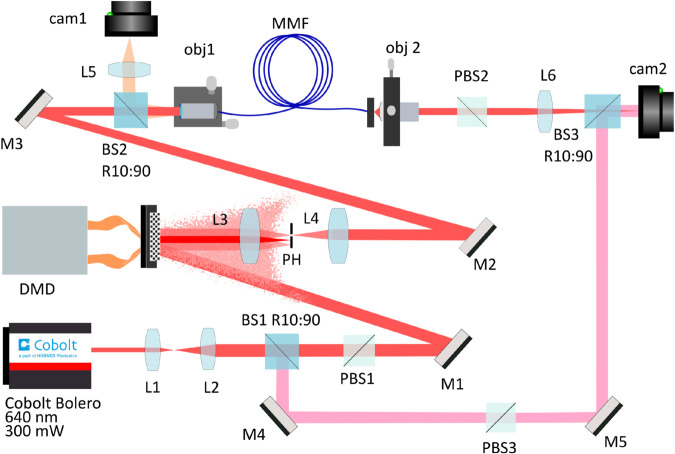
The schematic diagram of the experimental setup. The laser beam splits with BS1 directing 10% of the light towards mirror M4 to create a reference arm. The transmitted beam hits the DMD to generate multiple illuminations. Subsequently, the beam is coupled into the input facet of the MMF by OBJ1. The beam from the output facet is collected by OBJ2 and imaged on CAM2 using a tube lens L6. CAM1 is used to display the input facet of the MMF.

A detailed picture of the experimental setup is presented in Fig 1. The input facet of the MMF is scanned with a focused laser beam with a diffraction-limited spot size, where *d*_*lim*_ = 0.8 μm, to characterize the fiber. The beam is from a single-frequency laser source (Cobolt Bolero) with a wavelength of λ=640 nm and a maximum power of 300 mW. The light source has a specified linewidth of approximately 500 kHz, corresponding to a coherence length on the order of 600 meters. Only vertically polarized light filtered with a polarization beam splitter (PBS1, Thorlabs PBS251) was used to perform further measurements. The laser beam was expanded by a telescope (L1, Thorlabs, AC254-040-A-M; L2, Thorlabs, AC254-125-A-M) to increase the area that hits the DMD with a magnification of 3.12× to  4 mm diameter. The DMD (Texas Instruments), driven by the DLP9001VIS module (VIALUX), with 2560×1600 micromirrors and a pitch size of 7.6 μm, was used. DMD works like a 2D diffraction grating, so the light that is diffracted has many diffraction orders. One of these orders is isolated using the pinhole (PH, Thorlabs, SM1D12C) placed in the Fourier plane of L3. The 4f-system with two lenses (L3, L4, Thorlabs, AC508-150-A-ML-Ø2) was set to image the DMD on the back focal plane of objective OBJ1 (Olympus 20×, NA = 0.25). OBJ1 was used to couple the light into the MMF. By using camera CAM1 (IDS, U3–3880CP-M-GL, pixel size of 2.4 × 2.4 μm, 8 bit), the input facet of the MMF is imaged for alignment purposes.

For all measurements, a step-index MMF (Thorlabs, FG050UGA) with a core radius, a = 25 μm, NA = 0.22, and a length of 30 cm was used. The number of guided modes, M, can be estimated as $\text{M}=V^{2}/2$, where *V* is the normalized frequency, V=2πaNA/λ. The given MMF supports M≈1450 modes in total, or about 725 modes per polarization. After propagation through the fiber, the light is collected by objective OBJ2 (Leica 40×, NA = 0.65). The MMF output facet is imaged in camera CAM2 (IDS, U3-3070CP-M-GL, pixel size 3.45 μm). Inside the MMF, polarization mixing occurs, and PBS2 (Thorlabs PBS251) is used to separate the vertical polarization, ensuring control and measurement of the same polarization state. Sect 2.3 provides a detailed explanation of the implementation of off-axis and in-line holography methods.

### Off-axis and in-line holography

The basic principle behind holographic reconstruction is recording interference patterns created between a reference beam and an object beam. These two beams, which are coherent with each other, combine to create an intensity distribution that contains information about the phase and amplitude of the object beam. Off-axis holography is a phase imaging technique in which the reference and object beams overlap at a small angle [32]. An interference intensity profile, *I*(*x*,*y*), can be expressed as:

$$I(x,y)=|O(x,y){|}^{2}+|R(x,y){|}^{2}+O^{*}(x,y)R(x,y)+O(x,y)R^{*}(x,y),$$ (2)

where *R*(*x*,*y*) and *O*(*x*,*y*) denote reference and object fields, respectively.

The complex field *C*(*x*, *y*) = *O*(*x*, *y*)R^*^(*x*, *y*) can be recovered from the experimentally measured *I*(*x*,*y*). In our setup, the beam splitter, BS1 R10:90 (Thorlabs, BS025), deflects a portion of the beam for the off-axis holography reference arm. The mirrors M4 and M5 direct the reference beam towards camera 2. To characterize the TM across hundreds of MMF modes, a series of input illuminations was projected onto the DMD. We defined the working area of the DMD as 480×480 pixels, divided into 900 segments with the size of 16×16 micro-mirrors. A binary device, such as a DMD, creates a sinusoidal grating by applying the Lee holography method [33]. First, the desired wavefront is converted into a binary hologram using the Lee method, where the phase and amplitude of the wave are represented by the position and width of the fringes. Each binary hologram is then formatted according to the pixel grid (segment) of the DMD. For the experiments, a DMD segment contains a sinusoidal grating with an encoded phase pattern (φ=0). All other segments are turned off. This means that only one segment reflects the beam towards the MMF, while the remaining segments deflect the wavefront.

**Fig 2:**
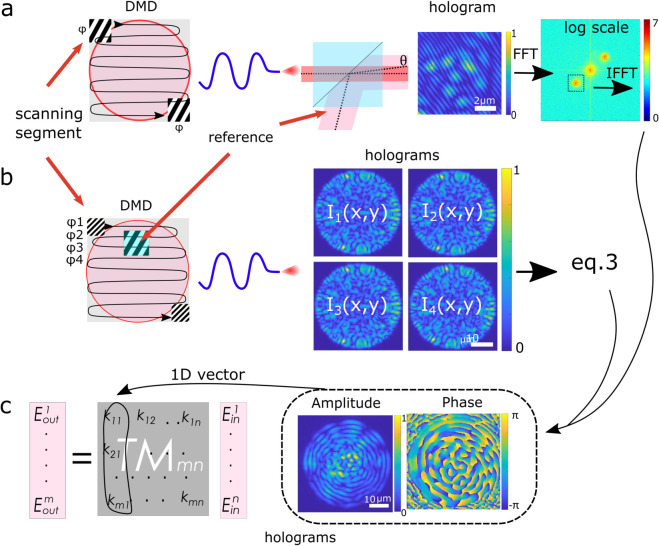
Schematic of complex field retrieval from holographic measurements. (a) Off-axis holography. Left: example DMD pattern where a single segment is active, set constant phase, and is changing the position across the DMD active area. Middle: resulting interference pattern of the MMF output and the reference plane wave. Right: sideband isolation in the Fourier domain and inverse transform to retrieve the complex field. (b) In-line holography. Left: DMD pattern showing a static internal reference region placed within the DMD working area and a modulated signal segment. The stack of resulting intensity patterns *I*(*x*,*y*,*n*) is processed to extract the complex output field. (c) From right to left: complex-valued output images corresponding to different input modes are flattened and form a TM.

By sequentially activating the segments, we scan the input facet of the fiber as illustrated in Fig 2(a) and obtain a set of interference patterns on camera 2. A total of 900 interference patterns were recorded, which formed a stack of measurements over the spatial domain. The complete stack can be expressed as: $ℐ(x,y,n)=\{I_{1}(x,y),I_{2}(x,y),\ldots ,I_{900}(x,y)\}$, where ℐ(x,y,n) is a three-dimensional dataset with dimensions corresponding to the spatial coordinates (*x*,*y*) and the image index *n*. By applying the Fourier transform to ℐ(x,y,n), the cross-terms can be isolated as distinct sidebands in the frequency domain, as shown in Fig 2(a). After selecting one sideband using a spatial filter mask, the inverse Fourier transform is applied to retrieve the complex field, from which the amplitude and phase information are extracted. Now, the complex-valued image matrices can be reshaped into the TM as shown in Fig 2(c).

In-line holography uses a common beam path for both the reference and signal beams, reducing the complexity of the optical setup and alignment requirements. It generally provides a stability advantage over off-axis holography, as the reference and signal beams share the same optical path. This minimizes relative phase drift caused by vibrations or environmental disturbances. In-line holography is a technique that involves creating a series of interference patterns using a predefined object beam with phase shifts.

In our implementation, the reference beam is generated internally by activating a small 20×20 micromirror region within the 480×480 active area of the DMD. This patch provides a fixed, known phase reference set to “0”, as shown in Fig 2(b). The remaining DMD segments sequentially scan the object field across the full working area; during this process, the scanning segment (16×16 micromirrors, 122×122μm) partially overlaps the reference region but never fully covers it, ensuring that the reference is always present in the recorded interference pattern. Because both reference and object fields co-propagate through the MMF, they experience identical modal dispersion and mode mixing. At the fiber output, their interference produces a speckle pattern, from which the complex optical field is reconstructed using the four-step phase-shifting method [34]. However, since the reference itself is scrambled by the MMF, it emerges as a random speckle field rather than a smooth wavefront, with a highly non-uniform and spatially varying phase.

Unlike off-axis holography, where each DMD segment has a fixed phase, in the in-line case, the relative phase shift between the reference and control sections is varied in four steps: $\phi_{1}=0$, $\phi_{2}=\pi /2$, $\phi_{3}=\pi$, and $\phi_{4}=3\pi /2$. For each of the 900 input positions, four interference patterns were recorded, resulting in a four-dimensional dataset: $ℐ(x,y,n,p)=\{I_{n,p}(x,y)\},$ where (*x*,*y*) are the spatial coordinates, image index *n*, and phase step index *p*. The “four-phase method” was used to retrieve the amplitude and phase. The complex field *C*(*x*,*y*) can be reconstructed as:

$$C(x,y)=\frac{I_{1}(x,y)-I_{3}(x,y)}{4}+i\frac{I_{4}(x,y)-I_{2}(x,y)}{4}$$ (3)

### Numerical simulations

To simulate light propagation through the MMF, a semi-analytical scalar mode solver was implemented under the weak-guidance approximation [35]. The simulation consists of two steps: calculation of all guided linear-polarized (LP) modes and their propagation constants, and modal decomposition and propagation of an arbitrary input field. For an input field $E_{in}(x,y)$, the output field after a fiber length *L* is given by:

$$E_{out}(x,y,L)=\sum_{n=1}^{N_{modes}}\alpha_{n}\;E_{n}(x,y)\;e^{-i\beta_{n}L},$$ (4)

where *E*_*n*_(*x*, *y*) is the transverse profile of the *n*-th guided LP mode, $\beta_{n}$ is its propagation constant, and $N_{modes}$ is the total number of guided modes. The coupling coefficients

$$\alpha_{n}=\iint E_{in}(x,y)\;E_{n}^{*}(x,y)\;dx\;dy$$ (5)

represent the overlap between the input field and each mode [36].

The guided-mode set $\left\{E_{n},\beta_{n}\right\}$ is obtained by solving the characteristic equation for a circular step-index fiber core:

$$\frac{u\;J_{m+1}(u)}{J_{m}(u)}=\frac{w\;K_{m+1}(w)}{K_{m}(w)},$$ (6)

with

$$u=a\sqrt{k_{0}^{2}n_{core}^{2}-\beta^{2}},\;w=a\sqrt{\beta^{2}-k_{0}^{2}n_{clad}^{2}}.$$ (7)

Here *a* is the core radius, $n_{core}$ and $n_{clad}$ are the core and cladding refractive indices, $k_{0}=2\pi /\lambda$ is the vacuum wavenumber, *J*_*m*_ and *K*_*m*_ are Bessel and modified Bessel functions of order *m*, and *m* is the azimuthal index. The quantities *u* and *w* are dimensionless transverse wave-number parameters. Once the modal basis is obtained, arbitrary input fields are propagated using Eq 4.

Both in-line and off-axis holography are simulated using the same mode solver and identical fiber parameters and spatial basis. A single input basis field is a 16×16 pxls (1.7×1.7μm) segment at the fiber input facet. The output field is sampled on a 480×480 pixel frame. TM${\;\;}_{\text{off}}$ is obtained by propagating each input segment with “0” phase through the MMF and extracting the complex output fields directly, corresponding to an ideal, noise-free off-axis measurement. For the in-line simulation, each input basis segment is sequentially encoded with four phase shifts (0, π/2, *π*, 3π/2), and each pattern is propagated through the MMF. The resulting noise-free intensity distributions at the MMF output are calculated. The complex field is reconstructed using Eq 3, and the resulting complex fields for all basis vectors constitute TM${\;\;}_{\text{in}}$.

Once TM${\;\;}_{\text{off}}$ and TM${\;\;}_{\text{in}}$ are obtained, all subsequent steps are identical for both methods. The desired output pattern (target, 480×480 pixels) is vectorized, multiplied by the conjugate transpose of the respective TM to compute the required phase-only input field, which is numerically propagated through the MMF to obtain the final output intensity distribution.

## Results

### Stability characterization

Wavefront engineering on the MMF output requires high interferometric stability. The in-line configuration provides inherent stability due to its common-path geometry, whereas the off-axis approach relies on an external reference and is therefore more sensitive to vibration and air turbulence. To quantify these effects, stability measurements were performed for both configurations using the same optical setup and a fixed acquisition rate of 120 Hz for off-axis and 30 Hz for in-line holography, respectively. To allow a direct comparison between the two phase-retrieval methods, the stability traces are evaluated over the same time window of 7.5 s.

**Fig 3:**
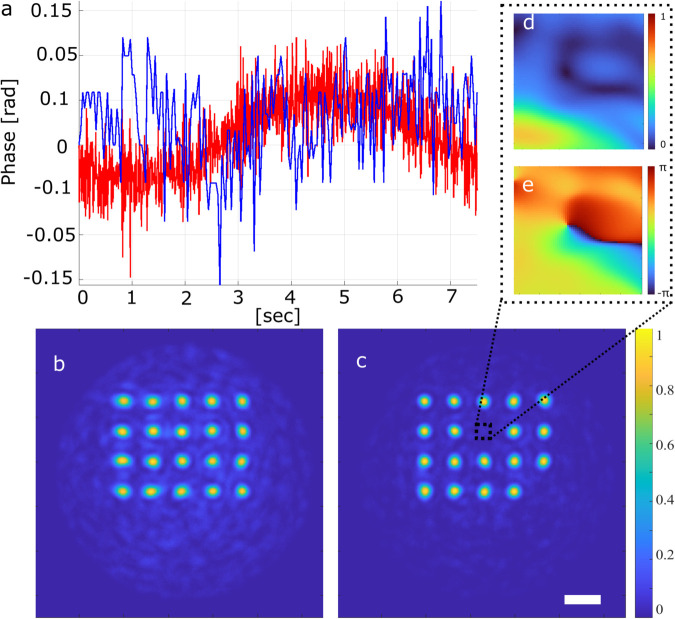
Experimental results. (a) Temporal phase stability of a single speckle grain at the MMF output measured over a 7 s interval for the in-line (blue) and off-axis (red) configurations. (b,c) Incoherent sum of 20 images of focal spots at the MMF output facet optimized with off-axis (b) and in-line (c) approaches. The scale bar is 10 μm. (d,e) Reconstructed intensity (d) and phase (e) distributions of the zoomed-in region (2×2 μm) corresponding to the singularity.

For in-line stability measurements, a sequence of four phases was projected in a single input position of the DMD segment, which is described in the Methods section. A separate DMD segment has been used as a zero-phase reference. All other DMD segments were switched off. A total of 3600 intensity patterns on the MMF output was recorded. The reconstruction procedure described above was applied to extract 900 light fields (both phase and amplitude). The area in pixels selected for analysis corresponds to the diffraction limit of the MMF, which is $d_{lim}=\lambda /(2\;NA)\approx 1.45\;\mu$m. The phase mean value with the 1.45 μm × 1.45 μm region was calculated as a function of time. Temporal phase fluctuations in a diffraction-limited region at the MMF output, measured using in-line holography, are shown in blue in Fig 3(a). The standard deviation of 900 phase measurements in 30 seconds is calculated as 0.03 radians.

A similar measurement procedure is applied for off-axis holography. A constant zero phase was projected onto the same input position of the DMD segment. All other segments were turned off. A total of 900 interference patterns between the field at the fiber output and the external reference have been recorded. The phase profiles have then been calculated as discussed in the Methods section. The standard deviation of 900 phase measurements over 7 seconds is equal to 0.04 radians. The results are presented in Fig 3(a) (red curve). Although in-line holography measurements have the potential for greater stability, our results show very similar performance for both the in-line and off-axis approaches.

### Projecting single-mode targets

To control the intensity distribution at the MMF output, the first experiment focused on generating a “single-mode” projection with a size of the diffraction-limited spot. A target field $E_{\text{out}}$ was defined as a 0.345×0.345 μm region (a 4×4 pixel area) at the desired camera-plane position. The corresponding input field was obtained by backpropagating the target through the conjugate transpose of the measured TM:

$$E_{\text{in}}={𝐓}^{†}E_{\text{out}}.$$ (8)

The chosen target area is much smaller than the diffraction limit of our MMF (1.45 μm), ensuring that the desired spot occupies a single spatial mode.

To quantify focusing performance, a power ratio (PR) metric was introduced, defined as the ratio of the intensity within the focal spot at its half-maximum level, $I_{\text{foc}}$, to the total transmitted intensity, $I_{\text{total}}$:

$$\text{PR}=\frac{I_{\text{foc}}}{I_{\text{total}}}$$ (9)

where I${\;\;}_{\text{foc}}$ is calculated by integrating the signal within a circular area with a radius equal to the full width at half maximum (FWHM) of the theoretical diffraction limit of the MMF.

First, we measure the full TM of the MMF using the off-axis holography approach. The TM${\;\;}_{\text{off}}$ consists of 419904×900 elements, where 419904 represents the size of the flattened camera image of 648×648 pixels, and 900 corresponds to the number of DMD segments. A set of 20 phase masks is used to sample different locations within the fiber core, each mask producing a single diffraction-limited focal spot after propagation through the MMF. Each phase mask is projected onto the DMD, and the resulting intensity at the MMF output facet is recorded. The incoherent sum of 20 individual focal spots is presented in Fig 3(b). The average PR of focal spots calculated using off-axis TM measurements is PR${\;\;}_{\text{off}}=41.4\pm 2.6\%$. The theoretical limit of PR corresponds to a perfect diffraction-limited focus, assuming ideal wavefront control and no background noise or mode-mixing artifacts. Although our approach did not achieve the theoretical maximum PR, previous work [26] has demonstrated that PR=96% is achievable, providing a reference to optimize imaging performance under various experimental conditions.

The same experimental setup, with the reference arm blocked, is used to measure the TM${\;\;}_{\text{in}}$ (419904×900) of the MMF via in-line holography. A set of 20 phase masks is then retrieved, each corresponding to an optimized diffraction-limited focal spot at predefined positions on the MMF output facet. Each mask is projected onto the DMD, and the resulting intensity patterns at the MMF output are recorded. The incoherent sum of 20 individual projections is presented in Fig 3(c). The average PR of the focal spots optimized using in-line TM measurements, PR${\;\;}_{\text{in}}=56.4\pm 17.8\%$.

The large standard deviation is primarily caused by phase singularities in the reference field, at points where the amplitude vanishes and the phase becomes undefined. At these locations the interference contrast collapses, preventing reliable phase retrieval. The resulting “blind spots” visible as dark speckle regions in the zoomed-in images in Fig 3(d), 3(e) degrade the fidelity of the TM, particularly for specific rows corresponding to those affected regions. This effect is a known limitation of in-line holography with speckle references, but it can be reduced by using multiple internal references or advanced calibration procedures [28,37], which improve focus quality and suppress the influence of blind spots. Excluding these “blind-spot regions”, in-line holography achieves a power ratio PR${\;\;}_{\text{in}}=61.8\;\pm \;6.5\%$, which is approximately 50% higher than the value obtained for off-axis holography under the same experimental conditions.

### Projecting complex freeform targets

**Fig 4:**
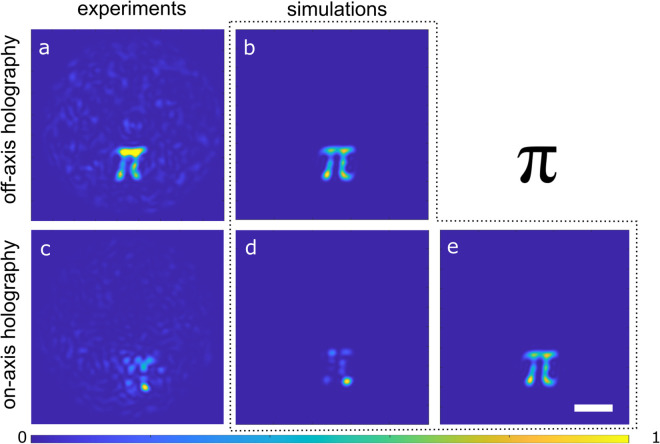
Intensity distribution created at the output facet of the MMF: experimental (a, c) and simulated (b,d,e) data. Results were obtained using off-axis (a,b) and in-line (c,d,e) holography. The binary target is shown in the top right. (d, e) Simulated result for in-line holography without (d) and with (e) reference field correction. The scale bar is 10 μm.

In the second set of experiments, we investigate the projection of complex, arbitrary-shaped targets through an MMF using different approaches to measuring TM. A binary *π*-shaped intensity distribution was chosen as the desired output pattern, and the corresponding optimized input fields were computed using the experimentally measured transmission matrices TM${\;\;}_{\text{off}}$ and TM${\;\;}_{\text{in}}$. The resulting output intensities are shown in Fig 4. Using TM${\;\;}_{\text{off}}$, the projected pattern (Fig 4a) closely reproduces the target shape and maintains clear boundaries. In contrast, projection using TM${\;\;}_{\text{in}}$ (Fig 4c) fails to recover the structure of the *π*-shape: the output lacks continuity and contains severe distortions. This result is markedly different from the performance observed for a diffraction-limited spot projection.

The non-homogeneous intensity distribution and rapidly varying phase of the in-line reference results in a discontinuous shape. Rapid phase changes within the target area, which in the case of complex shapes can be much larger than the diffraction limit, also disrupt the image projection. This results in phase discontinuities across the reconstructed field, making it impossible to reproduce large-scale structures.

Numerical simulations introduced in Sect 2.4 confirm these observations. The simulated projections are shown in Fig 4(b), 4(d). Wavefront shaping based on TM${\;\;}_{\text{off}}$ produces an accurate result, while TM${\;\;}_{\text{in}}$-based approach fails. This agreement between experiment and simulation demonstrates that the performance difference arises from the TM acquisition procedure itself, not from experimental noise or instabilities.

The off-axis simulation with TM${\;\;}_{\text{off}}$ produces a clear and accurate reconstruction, while the image projected with the in-line TM${\;\;}_{\text{in}}$ has no distinct boundaries or clearly defined shape. This agreement between experiment and simulation demonstrates that the performance difference arises from the TM acquisition procedure itself, not from experimental noise or instabilities.

To mitigate the limitations of the in-line holography, we applied a reference-field normalization to TM${\;\;}_{\text{in}}$, correcting the measured object field *C*(*x*,*y*) using the amplitude and phase of the reference field *R*(*x*,*y*):

$$O(x,y)=\frac{C(x,y)}{\left|R(x,y)\right|}\;e^{i\phi_{R}(x,y)}.$$ (10)

Fig 4(e) shows the simulated target projection after applying the reference-field correction to TM${\;\;}_{\text{in}}$. Once the influence of the non-uniform internal reference is removed, the in-line method produces an output pattern that closely matches the result of the off-axis approach (compare Fig 4(b), 4(e)). This convergence is expected, as both methods ultimately operate under the same physical constraints: the use of phase-only modulation, the finite number of guided modes supported by the MMF, and the diffraction limit imposed by the fiber’s NA. However, the correction requires independent measurement of the complex reference field, which removes the experimental simplicity and stability associated with in-line holography.

To quantify the fidelity of the target projection, the Pearson correlation coefficient (PCC) was used,

$$r=\frac{\sum_{i}(T_{i}-\bar{T})(R_{i}-\bar{R})}{\sqrt{\sum_{i}(T_{i}-\bar{T}{)}^{2}}\sqrt{\sum_{i}(R_{i}-\bar{R}{)}^{2}}}$$ (11)

which measures similarity between the target intensity *T* and the projected intensity *R*, *i* is the index of all pixels within the chosen ROI. Experimentally, TM${\;\;}_{\text{off}}$ achieved *r* = 0.51, whereas TM${\;\;}_{\text{in}}$ produced only *r* = 0.15. Simulations followed the same trend, yielding *r* = 0.82 for TM${\;\;}_{\text{off}}$ and *r* = 0.67 for TM${\;\;}_{\text{in}}$. After correction, TM${\;\;}_{\text{incorr}}$ improved to *r* = 0.79, confirming that the primary source of distortion in the in-line projections is the spatial structure of the internal reference.

In summary, off-axis holography enables accurate projection of complex freeform targets through an MMF, while in-line holography suffers from intrinsic reference-related distortions that prevent coherent reconstruction of extended shapes. Although reference correction can partially restore performance, TM${\;\;}_{\text{off}}$ measurements remain the more robust approach for complex pattern generation.

## Discussion

To further investigate the relative performance of in-line and off-axis holography for projecting complex targets through an MMF, we performed a systematic numerical study using a set of 10 grayscale MNIST digits (0-9). We simulated off-axis and in-line TM measurements using identical MMF parameters (core diameter 50 μm, NA=0.22, fiber length 1 m) as described in Sect 2.4. Projection fidelity was quantified using PCC (Eq 11), evaluated within a fixed region of interest (ROI).

**(i) Number of controlled modes.** The first set of simulations examined how the projection quality changes with the number of controlled DMD segments. Grayscale digits 1, 3, 5, 7, and 9 with a size of 20×20 μm positioned at the center of the fiber facet were used as targets. For each digit, two optimized input phase patterns were calculated using TM${\;\;}_{\text{in}}$ and TM${\;\;}_{\text{off}}$, respectively. We repeated simulations for different total numbers of controlled input segments from 100 to 1600.

**Fig 5:**
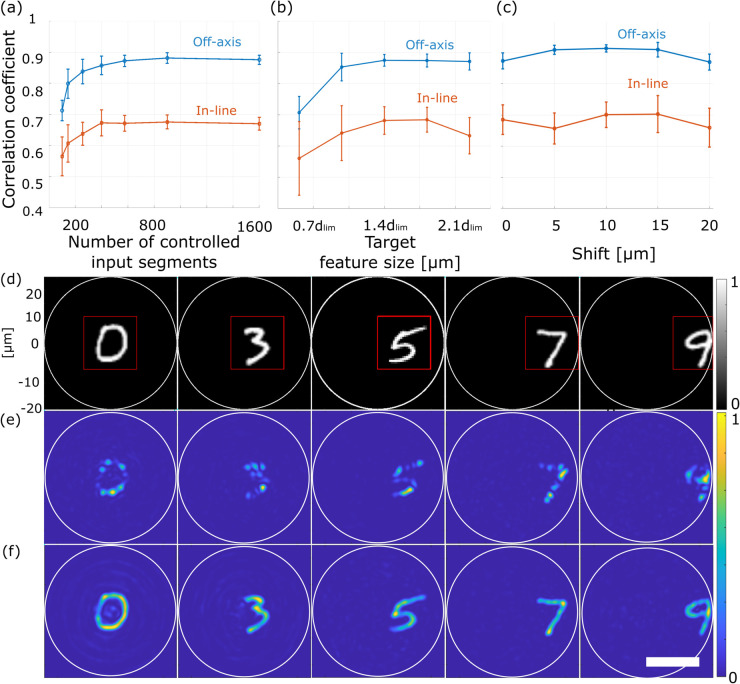
Systematic comparison of in-line and off-axis holography methods for wavefront shaping through an MMF for the MNIST dataset images as targets. (a-c) PCC between the target and projected images as a function of the number of controlled input segments (a); the target feature size (b); lateral target displacement across the fiber facet (c). $d_{lim}$ denotes the diffraction-limited spot size of the MMF. (d) Examples of target intensity patterns. The target position is shifted from the center from 0 to 20 μm in 5 μm steps (from left to right). The red square indicates the 20×20 μm ROI used for PCC evaluation. (e, f) Intensity distributions on the MMF output facet obtained by wavefront shaping with TM${\;\;}_{\text{in}}$ (e) and TM${\;\;}_{\text{off}}$ (f). The white circles indicate the MMF core area. Scale bar: 20 μm.

The results are presented in Fig 5(a). As the number of controlled segments increases, the correlation between the desired target shape and actual projection rises and eventually saturates once the number of controlled segments approaches the fiber’s guided-mode capacity, which is ∼1450 guided modes. Off-axis holography achieves r=0.87±0.02, whereas in-line holography saturates near r=0.67±0.02.

**(ii) Target feature size.** In the second set of simulations, we evaluated how target projection performance using TM${\;\;}_{\text{in}}$ and TM${\;\;}_{\text{off}}$ methods depends on the feature size of the target. We used 1600 controlled segments and ten grayscale MNIST digits (0–9) placed at the center of the MMF output facet as targets. We repeated simulations for different target sizes varied from $0.5\times d_{lim}$ to $2.0\times d_{lim}$, and ROI scaled proportionally (8×8 μm to 32×32 μm). The effective feature size of each digit was quantified using the FWHM of its normalized autocorrelation. $C(m,n)=\sum_{i}\sum_{j}A(i,j)\;A(i+m,\;j+n),$ where *i* and *j* enumerate pixel coordinates of the images, and (*m*,*n*) denote the spatial shifts.

The results are presented in Fig 5(b). For feature sizes below the diffraction limit $d_{lim}$, both methods yield low correlation. Once the feature size exceeds $d_{lim}$, the fidelity increases sharply. Off-axis holography reaches r=0.87±0.02, while in-line holography reaches only r=0.68±0.05.

**(iii) Target position across the fiber facet.** To evaluate how projection quality varies across the fiber facet, the 20×20 μm targets were shifted laterally five times in 5 μm steps from the center of the fiber core toward the cladding, as presented in Fig 5(c). For each position, the optimized input phase mask was calculated. The corresponding intensity patterns at the MMF output obtained using wavefront shaping with TM${\;\;}_{\text{in}}$ and TM${\;\;}_{\text{off}}$ are presented in Fig 5(d) and 5(e), respectively. As shown in Fig 5(c), the off-axis method maintains high and stable performance with a slight reduction at the center and near the cladding. The in-line method shows lower performance and higher noise

**(iv) Grayscale vs. binary targets.** Finally, we tested the performance quality for binary and grayscale targets. Optimized phase masks were calculated for grayscale and binarized versions of five MNIST digits (1–5) projected at the center of the fiber output facet. The performance of off-axis holography shows r=0.88±0.02 and r=0.84±0.04 for grayscale and binary targets, respectively. The in-line method followed the same trend but with lower fidelity of r=0.66±0.06 and r=0.63±0.07. Grayscale targets consistently performed better because their smoother intensity variations contain fewer high-frequency components, making them easier to reproduce and less sensitive to noise.

Overall, off-axis holography demonstrates better performance in projecting a variety of complex targets at multiple locations at the MMF output facet, whereas the in-line method generally suffers from random phase variations. We note that simulations demonstrate better performance than experimental results, as shown in Fig 4. The numerical model assumes ideal, noise-free conditions and the same linear polarization at the input and output of the MMF, whereas in the experiments, light scrambling leads to two orthogonal polarizations at the MMF output. Because we control only one, the wavefront shaping quality is reduced.

## Conclusion

Our findings reveal a key trade-off in wavefront shaping through an MMF using TM characterization: while in-line holography provides better focus quality and stability for single-mode diffraction-limited targets, off-axis holography excels in projecting complex-shaped patterns. In-line holography benefits from a common-path geometry, where both signal and reference fields co-propagate through the same MMF. This configuration ensures minimal phase drift and excellent stability. The issue of phase singularities in the internally generated reference field is a well-known limitation and has been addressed in prior work using multiple internal references. These techniques can mitigate “blind spots” and enhance single-spot focusing efficiency. However, even when singularities are avoided, the inherently non-uniform and rapidly varying phase of the reference field prevents globally consistent phase retrieval. Consequently, in-line holography cannot reliably reproduce extended or complex-shaped targets.

Off-axis holography, in contrast, uses a spatially flat external reference that bypasses the fiber and avoids modal scrambling. However, this approach comes with trade-offs: the optical setup is more complex, requiring separate reference and object paths, which increases sensitivity to environmental disturbances such as vibrations and air turbulence. The off-axis reference, traveling outside the MMF, picks up independent phase noise. These independent phase fluctuations introduce temporal phase noise, which is especially detrimental when projecting diffraction-limited spots, where precise phase alignment is essential for optimal focusing. As a result, off-axis holography, while ideal for projecting complex targets, is inherently less stable for single-spot generation than the in-line configuration. Understanding this trade-off is essential for optimizing MMF-based imaging, beam shaping, and signal delivery across applications in metrology, telecommunications, and biomedical optics.

## Supporting information

S1 Table Raw data for stability measurements presented in Fig 3(a). (CSV)

S2 Table Correlation coefficient as a function of the number of controlled input segments presented in Fig 5(a). (CSV)

S3 Table Correlation coefficient as a target feature size presented in Fig 5(b). (CSV)

S4 Table Correlation coefficient as a function of the target displacement across the fiber facet presented in Fig 5(c). (CSV)
